# Poverty in the Brazilian Amazon and the challenges for development

**DOI:** 10.1590/0102-311XEN100223

**Published:** 2023-10-13

**Authors:** Danuzia Lima Rodrigues, Daniel Nogueira Silva

**Affiliations:** 1 Universidade Federal do Sul e Sudeste do Pará, Marabá, Brasil.; 2 Universidade Federal do Pará, Marabá, Brasil.

The economic and social transformations that occurred in the Brazilian Amazon in the 20th century show the profound contradictions of the development models implemented in the region throughout its history. The natural resources mapped in the region since the 1950s, some of them identified before that, have placed this territory at the center of national and regional political disputes for the exploitation of its wealth ^1^. With the advance and appropriation of these resources by large capitalist companies, evidence indicates that these models of development do not contribute to better living conditions for the local populations.

Data ^2^ on the region expose a set of needs and vulnerabilities to which populations in the Amazon are subjected. The classic indicators of poverty, such as the Municipal Human Development Index (M-HDI) and per capita income, indicate that the Amazon remains one of the poorest regions in Brazil. In 2020, the average M-HDI of the municipalities in the region was 0.736, which is considered high but still below the national average. Average monthly income of employed persons aged 14 years or older in the region was BRL 2,059.75 in 2020, also below the national average of BRL 2,782.5. According to the Brazilian Institute of Geography and Statistics (IBGE), in 2019, about 20.9% of the population in the region lived below the poverty line, that is, with a monthly per capita income less than BRL 486.00. About 9.7% of the population lives in extreme poverty, with per capita income up to BRL 168.00. As a consequence, despite accounting for only 8.6% of the country’s population in 2019, this territory presented 15.73% of the total Brazilian population living in poverty and extreme poverty.

Access to basic sanitation is another factor that increases vulnerability in this territory. In 2021, according to the Brazilian National Sanitation Information System (SNIS) ^3^, almost 40 and 80% of the population did not have access to drinking water and sanitation services, respectively. According to a large body of literature ^4^, the scarcity of drinking water and the lack of adequate sanitation services contribute to the spread of water-borne diseases and increase the risk of contamination and infection. Considering that access to work opportunities and income is linked to health conditions, based on a set of causal relationships, the lack of basic sanitation perpetuates cycles of poverty that are difficult to break ^5^.

This set of factors ^6^ exposes some of the limits of the development models underway in the Amazon. The economic dynamics generated by the exploitation of mineral resources and timber products, as well as by the production of soybean commodities and extensive livestock, bring positive economic indicators, such as the growth of gross domestic product (GDP) and exports, but neglect the deleterious social effects generated from these same activities ^7^. As an example, we can cite the fact that the main mining municipalities in the Amazon ^8^ still have significant portions of their population living in precarious urban conditions ^9^ and with low incomes ^10^, as indicated by the Unified Registry (*Cadastro Único*) ^11^ microdata, even though they yield a great economic wealth.

These classic poverty indicators emphasize the magnitude of the social and economic problem faced by the Amazon Region, reinforcing the need to create public policies that promote human and economic development, reducing deprivation and social vulnerability. In this sense, the ideas proposed by Amartya Sen ^12^, based on the Capability Approach, provide a useful framework to broadly analyze social vulnerability in the region. Based on its multidimensional approach to poverty, it is possible to identify the most vulnerable people and communities and understand the underlying causes of poverty in the region.

By considering a variety of indicators, such as education, health, housing, and access to basic services, studies based on the analysis of multidimensional poverty highlight the complex interconnections that perpetuate deprivation. Moreover, they reinforce the importance of integrated and targeted public policies to address the multiple dimensions of poverty. By specifically investigating rural and urban areas in Brazil, some of these studies bring crucial elements to understand regional disparities and the distinct dynamics that influence poverty ^13^. The results show that health and sanitation accounted for the greatest impact on multidimensional poverty, followed by education and housing conditions.

Assessing or measuring multidimensional poverty in the Amazon Region based on Sen’s ^12^ approach also offers important contributions ^14^ ^,^ ^15^ ^,^ ^16^. These studies highlight how the complex interaction between development and poverty can take particular forms in specific geographical contexts, such as that of the Brazilian Amazon. Moreover, they also contribute a more focused perspective, examining how poverty in specific contexts can affect other variables such as parenting and family relationships, further enriching the understanding of the complex implications of multidimensional poverty.

This holistic approach, complemented by the contributions of different surveys, offers a more complete picture of the socio-economic realities in Brazil, allowing for a more effective allocation of resources and efforts to substantially improve the living conditions of marginalized groups. However, in some contexts, capturing territorial particularities and dynamics by using variables commonly used in this literature cannot reflect the conditions of deprivation experienced by the inhabitants of a given territory. A study conducted by Rodrigues ^16^ using the methodology of the Multidimensional Poverty Index (MPI) of Alkire & Santos ^17^, which is based on Sen’s ^12^, illustrates this issue.

The field laboratory chosen by the author was a floodplain region, presenting characteristics of an island, and which can serve as a reference for further analyses since part of the Amazon territory is made up of an immense archipelago of river islands, inhabited by ribeirinho populations ^18^. The study analyzed the Ilhas das Onças, located in the Metropolitan Area of Belém, Pará State. Notably, this particular region shows a remarkable peculiarity: it is a major producer of açaí. Its proximity to the capital, Belém, facilitates the production flow, giving it a distinct economic dynamism compared to other areas that share similar characteristics. However, it is noteworthy that this region also faces the direct and indirect impacts of the growing process of urbanization throughout the area. The innovation of the research, which represents a leap forward in the literature that works with this methodology, lies in the identification and definition of dimensions and functions identified as priorities by the study population. It proposes to evaluate the development of human capacities considered relevant by the locals in the search for a good quality of life and to understand how the conditions of the territory allow or limit the expansion of the substantive freedoms that residents can obtain from the place where they live. In this way, the methodology helps to incorporate the priority needs identified by the communities themselves into the analysis.

The four versions of the MPI calculated in that study, including an adjusted version with dimensions and weights assigned by the interviewees, allow us to understand more specifically how the use of synthetic indicators constructed from fixed dimensions and weights can hide the vulnerabilities suffered and/or perceived by people. This difference becomes evident when comparing, for example, the different results of MPI. When the indicators and weights conventionally used in the literature are applied, the MPI indicates that 38% of the sample lives in multiple poverty. However, when adopting indicators and weights defined endogenously by the local population, this percentage drops to 31%. The same occurs when disaggregating the indices: the proportion of poor in multidimensional terms drops from 75% (exogenous) to 55% (endogenous).

These findings confirm the importance of considering what residents value in quality of life when measuring poverty. Evidence shows that when applying Sen’s ^12^ capabilities approach, the poverty indicator decreases if the dimensions weight are endogenously collected. This suggests that homogenizing poverty measures, disregarding peculiarities of each region, may result in an overestimation of poverty levels. A relevant aspect is the potential for directing public policies towards areas that may not actually improve local quality of life.

An interesting fact found by Rodrigues ^16^ - corroborating this view - can be observed when the interviewees were asked to spontaneously list the four most important dimensions. Among all dimensions, health was identified as the most important by 44% of respondents. However, even with the highest weight assigned, when applying the methodology, health was not the dimension that contributed the most to the formation of the indices, suggesting difficulties in choosing indicators capable of capturing the restrictions in relation to this dimension. This incongruity was later revealed by the absence of variables that could accurately capture what the local population considers important. Thus, variables and indicators generally used to evaluate this type of dimension failed to represent the main problem in the health dimension. It became clear during the study that the health problems that the interviewees referred to as the “most important” were directly linked to mobility and accessibility to emergency healthcare services, and therefore related to risks in the serious events.

In addition to improving the diagnosis of poverty and vulnerability in the studied territory, the results of the study point to the complexity of assessing multidimensional poverty when addressing a specific territoriality. The inclusion of aspects that people consider important, such as being or doing, reveals deprivations that are not normally considered by indices constructed with externally predetermined weights, such as synthetic indices. These elements reinforce the importance of considering the specific characteristics and demands of each region when formulating public policies to combat poverty and vulnerability, especially in the Amazon. This effort can contribute to the construction of tools and public policies that help promote more inclusive development models aimed at local demands.
